# Intraduodenal sarcoma recurrence of retroperitoneal origin: an unusual cause for a duodenal obstruction

**DOI:** 10.1186/1477-7819-10-59

**Published:** 2012-04-20

**Authors:** Jean J Bao, John C Mansour, Robert D Timmerman, Amanda Kirane, Gene E Ewing, Roderich E Schwarz

**Affiliations:** 1 Department of Surgery, Division of Surgical Oncology, UT Southwestern Medical Center, Dallas, TX, USA; 2 Department of Radiation Oncology, UT Southwestern Medical Center, Dallas, TX, USA; 3 Department of Pathology, UT Southwestern Medical Center, Dallas, TX, USA

**Keywords:** Intraduodenal sarcoma, radiation treatment, retroperitoneal sarcoma

## Abstract

Soft tissue sarcomas are uncommon tumors, and intraduodenal soft tissue sarcoma manifestation is even more rare. Only three cases of intraduodenal sarcomas have been reported in the literature thus far. Here, we report a case of an intraduodenal recurrence of a retroperitoneal sarcoma causing bowel obstruction. This unusual recurrence pattern likely relates to the patient’s previous resection and radiation treatment, and highlights the benefits, limitations and follow-up strategies after multimodality treatment.

## Background

Soft tissue sarcomas (STS) are rare tumors, accounting for less than 1% of all newly diagnosed malignancies [1]. Of all STS, only 20% originate from retroperitoneal and intra-abdominal locations [2]. These tumors are commonly extensive and locally invasive, rendering complete surgical resection challenging. Nonetheless, resection remains the mainstay of therapy for retroperitoneal and intra-abdominal STS, because radiotherapy is limited due to incomplete responses and potential toxicity to adjacent structures, and because highly effective adjuvant chemotherapy remains to be identified. To the best of our knowledge, there are only three cases of intraduodenal STS reported in the literature, two of which are recurrent retroperitoneal STS and one being synchronous with the retroperitoneal sarcoma [3-5]. We report a case of an intraduodenal recurrence of a retroperitoneal sarcoma causing bowel obstruction.

## Case presentation

A 60-year-old male patient had previously undergone a radical resection of a T2bN0M0G3 Stage III retroperitoneal sarcoma, including right nephrectomy and cholecystectomy, in 2002. Pathologic evaluation demonstrated a 25 cm high-grade liposarcoma with different histologic components, including myxoid, round cell, well-differentiated, sclerosing and pleomorphic patterns. Surgical margins were negative as the tumor did not invade the kidney, and since the renal vein and ureter were free of tumor as well.

The patient did not undergo any additional therapy. Surveillance magnetic resonance imaging in 2007 revealed an isolated tumor recurrence in the retroperitoneum. An exploratory celiotomy revealed tumor involvement of the duodenum, head of the pancreas, superior mesenteric vein, vena cava and left renal vein with severe adhesive changes, leading to the intraoperative assessment of unresectable disease. Subsequently, the patient underwent stereotactic body radiation to the retroperitoneal tumor in five fractions for a total dose of 30 Gy, without complication. Postradiation imaging revealed near complete resolution of the retroperitoneal mass.

**Figure 1:**
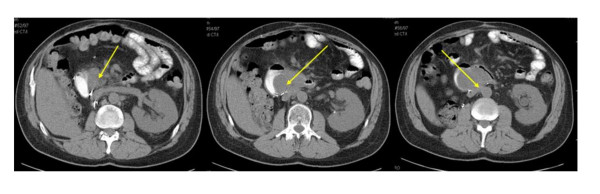
Recurrent tumor and relationship to the duodenum (left and center image) and large vessels (right image), as indicated by the arrows.

**Figure 2:**
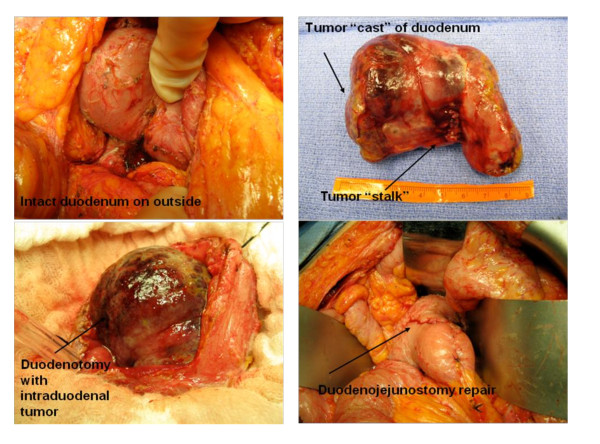
Intraoperative images of an entirely intraduodenal tumor resected with primary duodenojejunal repair.

One year later, the patient presented with weight loss, postprandial abdominal pain, nausea and vomiting. Cross-sectional imaging revealed a 7 cm mass with involvement of the third portion of the duodenum (Figure 1). However, there was no obvious involvement of the pancreatic head or the other structures that were noted to be involved during the previous laparotomy. We elected to perform another resection attempt, including a possible pancreatoduodenectomy or retroperitoneal vascular resection if necessary. At exploration, the residual tumor was an entirely intraduodenal, pedunculated mass at the posterior duodenal wall distal to the ampulla that filled the entire duodenal lumen. The patient underwent a duodenotomy and stalk transection of the polypoid mass, followed by partial duodenal resection with hand-sewn duodenojejunal anastomosis (Figure 2). Surgical pathology examination revealed a 9.5 cm recurrent high-grade liposarcoma with polypoid intraluminal growth containing myxoid, round cell, well-differentiated, sclerosing and focally pleomorphic areas. Surgical margins were negative as the tumor came within 0.1 cm of the stalk margin and there was no evidence of additional neoplastic components within the remaining resected duodenum. The postoperative course was uncomplicated, and the patient has demonstrated no recurrence up to 30 months from this resection.

## Discussion

Intraduodenal presentation of STS is rare. To the best of our knowledge, there are only three cases reported in the literature. In the patient described, duodenal involvement by the extraduodenal sarcoma may have developed from either contiguous extension or hematogenous metastasis. However, at the time of the duodenectomy, there was no evidence of any extraduodenal disease or any distant metastasis. The solely intraduodenal growth pattern suggests that the previous radiation therapy to the retroperitoneal area, with duodenal shielding, had an impact in generating such an unusual disease pattern. This duodenal shielding from radiation together with a complete extraduodenal response to radiation therapy may thus have facilitated the isolated intraduodenal recurrence.

Liposarcoma is the most common histologic subtype of retroperitoneal sarcomas, with a higher local recurrence rate than non-liposarcomas (58% versus 22%) [6]. Even when tumor grade is taken into account, failure of disease control is mostly due to locoregional recurrence. For liposarcomas with greater potential for positive margin resections or higher risk for recurrence, preoperative radiotherapy should be considered. Radiation therapy is frequently used as an adjunct to operative therapy in the treatment of retroperitoneal sarcomas, administered at a dose of 40 to 50 Gy. Tumors with a significant myxoid component are more likely to demonstrate a size reduction when treated with external beam radiation [7]. Higher doses can be associated with significant gastrointestinal toxicity, which is the reason for contouring of the radiation field to spare those structures most susceptible to radiation-induced injury, such as the duodenum [8]. Our patient received a dose of 30 Gy in five fractions which effectively controlled the extraduodenal disease. The dose to the duodenum was reduced by intensity modulation. As the planning target volume overlapped portions of the circumference of the duodenum, the adjacent duodenal wall was included in the high dose volume. An avoidance structure was contoured for planning consisting of the non-adjacent wall of the duodenum outside of the planning target volume. Strict avoidance criteria were placed on this structure allowing steep dose falloff gradients specifically in the direction of the duodenum. This specific intentional shielding of the duodenum is likely the factor responsible for the development of this unusual pattern of recurrence.

The patient was symptomatic from the duodenal recurrence with partial bowel obstruction and weight loss. Given his excellent performance status and the lack of vascular involvement by the tumor, the decision was made to resect the lesion. Not resecting the tumor would almost certainly have guaranteed tumor progression and, ultimately, oncologic failure with a high risk for death from the disease. Radiation was not considered as part of the therapy for the duodenal recurrence as there was clear evidence of bowel involvement. Performing radiation therapy on the bowel for a malignant obstructive process would likely lead to significant gastrointestinal toxicity, with questionable outcomes regarding the resolution of the obstructive component.

## Conclusions

Complex retroperitoneal STS often mandate a multidisciplinary approach to treatment sequencing and selection. An awareness of sarcoma subtype-specific responses to radiation may help select patients who are more likely to demonstrate extraordinary responses. For patients with initially unresectable tumors and good response to radiation therapy, resection options should be re-evaluated as they can lead to lasting disease or symptom control.

## Consent

Written informed consent was obtained from the patient for obtaining intraoperative images for educational or publication purposes. The authors submitted a waiver from their institutional review board (IRB) stating that this case report does not require IRB approval or oversight.

## Competing interests

The authors declare that they have no competing interests.

## Authors’ contributions

JB drafted and revised the manuscript. JM contributed to the content of the manuscript and critically reviewed the manuscript. RT contributed to the content of the manuscript and revised the manuscript critically for important intellectual content. AK contributed to the content of the manuscript. GE contributed to the content of the manuscript. RS conceived of the case report, contributed to the content of the manuscript, revised the manuscript critically, and approved the final version for publication. All authors read and approved the final manuscript.
